# N‐Acetyl‐L‐Cysteine (NAC) Blunts Axitinib‐Related Adverse Effects in Preclinical Models of Glioblastoma

**DOI:** 10.1002/cam4.70279

**Published:** 2024-10-08

**Authors:** Alessia Formato, Maria Salbini, Elisa Orecchini, Manuela Pellegrini, Mariachiara Buccarelli, Lucia Ricci Vitiani, Stefano Giannetti, Roberto Pallini, Quintino Giorgio D'Alessandris, Liverana Lauretti, Maurizio Martini, Valentina De Falco, Andrea Levi, Maria Laura Falchetti, Maria Patrizia Mongiardi

**Affiliations:** ^1^ Institute of Biochemistry and Cell Biology, IBBC‐CNR Rome Italy; ^2^ Department of Neuroscience, Neurosurgery Section Università Cattolica del Sacro Cuore Rome Italy; ^3^ Department of Oncology and Molecular Medicine Istituto Superiore di Sanità Rome Italy; ^4^ Department of Neuroscience, Institute of Anatomy Università Cattolica del Sacro Cuore Rome Italy; ^5^ Department of Neurosurgery Fondazione Policlinico Universitario A. Gemelli IRCCS Rome Italy; ^6^ Università degli Studi di Messina Messina Italy; ^7^ Institute of Endocrinology and Experimental Oncology (IEOS), National Research Council (CNR) Naples Italy

**Keywords:** axitinib, brain tumor xenograft, endothelium, glioblastoma IDH‐wild type, glioma stem cells, N‐acetyl‐L‐cysteine, therapy, toxicity

## Abstract

**Objective:**

Axitinib is a tyrosine kinase inhibitor characterized by a strong affinity for Vascular Endothelial Growth Factor Receptors (VEGFRs). It was approved in 2012 by Food and Drug Administration and European Medicines Agency as a second line treatment for advanced renal cell carcinoma and is currently under evaluation in clinical trial for the treatment of other cancers. Glioblastoma IDH‐wild type (GBM) is a highly malignant brain tumor characterized by diffusely infiltrative growth pattern and by a prominent neo‐angiogenesis. In GBM, axitinib has demonstrated a limited effectiveness as a monotherapy, while it was recently shown to significantly improve its efficacy in combination treatments. In preclinical models, axitinib has been reported to trigger cellular senescence both in tumor as well as in normal cells, through a mechanism involving intracellular reactive oxygen species (ROS) accumulation and activation of Ataxia Telangiectasia Mutated kinase (ATM). Limiting axitinib‐dependent ROS increase by antioxidants prevents senescence specifically in normal cells, without affecting tumor cells.

**Methods:**

We used brain tumor xenografts obtained by engrafting Glioma Stem Cells (GSCs) into the brain of immunocompromised mice, to investigate the hypothesis that the antioxidant molecule N‐Acetyl‐L‐Cysteine (NAC) might be used to reduce senescence‐associated adverse effects of axitinib treatment without altering its anti‐tumor activity.

**Results:**

We demonstrate that the use of the antioxidant molecule N‐Acetyl‐Cysteine (NAC) in combination with axitinib stabilizes tumor microvessels in GBM tumor orthotopic xenografts, eventually resulting in vessel normalization, and protects liver vasculature from axitinib‐dependent toxicity.

**Conclusion:**

Overall, we found that NAC co‐treatment allows vessel normalization in brain tumor vessels and exerts a protective effect on liver vasculature, therefore minimizing axitinib‐dependent toxicity.

## Graphical abstract

Schematic representation of the experimental approach. In GBM preclinical models, we observed the efficacy of NAC cotreatment in protecting vessels from axitinib‐induced toxicity.

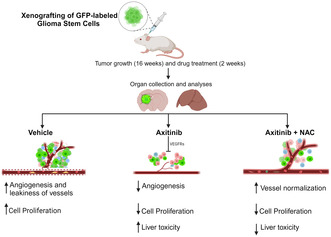

## 1 Introduction

Axitinib is a tyrosine kinase inhibitor with a high specific activity or vascular endothelial growth factor receptors 1, 2, and 3 (VEGFR1, 2 and 3). It is a small molecule with a MW of 386.47. Axitinib is effective on VEGFRs at picomolar concentrations, although, at higher concentrations (in the order of nanomolar), it inhibits PDGF receptors too [1]. When used at picomolar concentrations, axitinib specifically inhibits VEGF‐dependent receptor autophosphorylation allowing the inhibition of endothelial cells growth, survival, capability to migrate and to form tubes. The inhibition of VEGF impairs proliferation and vessel formation in in vivo tumor models [2]. Based on its efficiency in targeting VEGF pathway, axitinib has been proposed as a therapeutic option firstly for advanced renal cell carcinoma [3] and for colorectal cancer [4, 5]. Glioblastoma IDH wild type (GBM) is the most aggressive type of central nervous system (CNS) tumor in adults. In 2021, World Health Organization (WHO) updated the criteria for Classification of the CNS tumors. GBM is currently defined by diffusely infiltrative growth pattern with nuclear atypia and either (i) histologically identified by rapid mitotic activity and microvascular proliferation or necrosis; (ii) characterized at the molecular level by the presence of TERT (telomerase reverse transcriptase) promoter mutation, EGFR gene amplification and lacks of mutations in IDH1/IDH2 genes [6]. Standard treatment for GBM patients is based on the Stupp protocol which combines surgical intervention with adjuvant radio and chemotherapy with temozolomide (TMZ). A number of novel treatments have been proposed, some of which target VEGF [7] and its receptors [8, 9], leading to angiogenesis inhibition. Although axitinib effectiveness as a monotherapy in GBM patients is limited [10, 11], it exerts a stimulatory effect on T cells in recurrent GBM patients [12]. Interestingly, it was recently demonstrated that axitinib, in combination with the tricyclic antidepressant imipramine, contributes to significant therapeutic benefit in preclinical models of GBM mainly through activation of a potent immune response [13], strongly suggesting that axitinib might be an effective drug for GBM cotherapy. When considering axitinib for cancer therapy, it should be taken into consideration that it might be responsible for undesired side effects. Others [14, 15] and us [16] demonstrated that axitinib induces cell senescence in vitro. We showed that axitinib‐dependent senescence in endothelial cells requires oxidative stress increase and activation of the ataxia telangiectasia mutated (ATM) kinase [16]. Strikingly, prevention of axitinib‐dependent reactive oxygen species (ROS) increase through the use of antioxidants selectively avoids senescence induction of endothelial cells, with no effect on tumor cells, which invariably undergo senescence. We therefore hypothesized that if we coadminister antioxidants with axitinib, we might lower axitinib toxicity on endothelial cells, without affecting the drug effectiveness against cancer cells. Here we demonstrate that the use of the antioxidant molecule N‐acetyl‐cysteine (NAC) in combination with axitinib stabilizes tumor microvessels, eventually resulting in vessel normalization, and protects liver vasculature from axitinib‐dependent toxicity.

## 2 Materials and Methods

### 2.1 Cell Cultures

Glioma stem cells (GSCs) were isolated from GBM tumor resection as previously described [17, 18, 19, 20]. Briefly, undifferentiated tumor cells were isolated by mechanical dissociation of tumors resected from GBM patients. Isolated cells were maintained into a serum‐free culturing media, containing epidermal growth factor (EGF) and basic fibroblast growth factor (bFGF), as in [20]. As extensively described, these cells, although obtained as a heterogeneous cell population, maintained an undifferentiated state in culture. All patients signed an informed consent form allowing for collection of tumor samples and clinical data and for cell line generation. Cell cultures were regularly checked to exclude mycoplasma contamination by Mycoalert Detection Kit (Lonza, Basel, Switzerland).

Axitinib (AG‐013736, Sigma‐Aldrich, St Louis, MO, USA) was resuspended in DMSO.

N‐acetyl‐L‐cysteine (NAC) (A7250, Sigma‐Aldrich) was resuspended in PBS.

### 2.2 Cell Viability Assays

Cell Titer 96 Aqueous One Solution Cell Proliferation Assay (Promega, Madison, WI, USA) was used to evaluate the effect of axitinib treatment on GSCs and HeLa cells, as non‐GBM cell line, viability. Briefly, GSC#1, GSC#61, and HeLa were dissociated and plated on a 96‐well microplate in technical triplicate. Twenty‐four hours postplating, we added axitinib. MTS was performed 48 h later. Cell viability of axitinib‐treated cells was calculated as the percentage of vehicle‐treated cells. Axitinib concentration able to induce 30% of cell viability reduction (IC30) was chosen for subsequent experiments. Calcein AM staining has been performed on cell cultures to evaluate the percentage of viable cells. Cells were harvested, washed in PBS, and stained by Calcein AM 2 μM (Biotium, San Francisco, CA, USA) for 30 min. The percentage of stained cells has been counted by Attune NxT Flow Cytometer (Thermo Fisher Scientific, Waltham, MA, USA).

### 2.3 Western Blot Analysis

GSCs protein extracts were prepared in lysis buffer (50 mM TRIS–HCl pH 7.5, 250 mM NaCl, 1% NP‐40, 5 mM EDTA, 5 mM EGTA, 1 mM phenylmethylsulfonyl fluoride, 1 mM orthovanadate, adding a mix of protease inhibitors, Sigma‐Aldrich). For immunoblotting, 30 μg of proteins were separated by sodium dodecyl sulfate‐PAGE and transferred onto nitrocellulose membrane. All immunoblots were revealed by enhanced chemiluminescence (ECL) SuperSignal West Pico PLUS Chemiluminescent Substrate (#34580, Thermo Fisher Scientific). The antibodies used are: anti‐phopsho‐ERK 1/2 (#4370, Cell Signaling, Danvers, MA, USA; 1:1000), anti‐ERK 1/2 (#4695, Cell Signaling; 1:1000), anti‐Vinculin (#13901, Cell Signaling; 1:1000). Graphs report quantitation of the proteins levels as addressed by ImageJ software.

### 2.4 SA‐β‐Galactosidase Assay

For SA‐β‐galactosidase detection in GCSs, cells were harvested at the indicated time after axitinib treatment and gently dissociated. 1.5 × 10^4^ cells were transferred onto microscope slices by Thermo Shandon Cytospin 3 Centrifuge (Thermo Fisher Scientific), fixed in 3.7% formaldehyde for 5 min and washed in PBS with Ca/Mg. The slices were stained in a freshly prepared staining solution containing: 5‐bromo‐4‐chloro‐3‐Indolyl‐β‐D‐galacto‐pyranoside 1 mg/mL, citric acid/sodium phosphate buffer (pH 6.0) 40 mM, K_3_Fe(CN)_6_ 5 mM, K_4_Fe(CN)_6_ 5 mM, NaCl 150 mM, MgCl_2_ 2 mM, and placed at 37°C for 18 h (GSC#1) and 4 h (GSC#61). β‐galactosidase‐positive cells were visualized in bright field by Olympus BX41 DIC Microscope.

### 2.5 ROS Measurement

ROS content in GSCs cultures after axitinib treatment was assessed by 2′,7′‐Dichlorofluorescein diacetate (DCFHDA; Sigma‐Aldrich) staining. 1 × 10^5^ cells were harvested in HBSS, stained by DCFHDA 10 μM for 30 min at 37°C, and washed in HBSS. The evaluation of fluorescence was performed by Varioskan Instrument (Thermo Fisher Scientific). For ROS detection in ex vivo samples, the slices were stained in a solution containing dihydroethidium (DHE) 10 μM for 15 min, room temperature, and washed in PBS. The fluorescence signals have been visualized by Olympus AX70 Fluorescence Microscope.

### 2.6 Real Time PCR Analyses

Total RNA derived from GSCs was isolated by TRIzol (Thermo Fisher Scientific). cDNA was obtained using 1 μg of RNA and MLV reverse transcriptase (Promega) and amplified by Real‐Time PCR using SYBR Select Master Mix (Applied Biosystems, Foster City, CA, USA) and the QuantStudio 5 Real‐Time PCR System (Applied Biosystems). The primer sequences used are the following: laminB1 (*LMNB1*) (forward primer: GAAGAAGCAGCTGGA, reverse primer: TTGGATGCTCTTGGG) and TATA Box Binding Protein (*TBP*) (forward primer: TGCCCGAAACGCCGAATATAATC, Reverse primer: TGGTTCGTGGCTCTCTTATCCTC). Statistical analysis was performed using Prism software (GraphPad software) from three biological replicates.

### 2.7 3D Tumor Spheroids

GSC#1 and GSC#61 were suspended in complete growth medium containing 20% methylcellulose (Sigma‐Aldrich) (1 × 10^3^cells/spheroid in 100 μL) and plated on ULA round‐bottom 96‐well plates (Corning, Corning, NY, USA). After 48 h, spheroids were embedded into collagen matrix, Type I (Merck‐Millipore, St Louis, MO, USA) as indicated in [21], in a buffered solution containing bicarbonate to allow the formation of a solid three‐dimensional matrix. After 30 min, axitinib, NAC, axitinib+NAC, or vehicle were added. One week later, tumor spheroids were stained by Calcein AM 1 μM (Biotium), propidium iodide 4 μg/mL (Sigma‐Aldrich), and Hoechst 10 μg/mL (Sigma‐Aldrich), to detect metabolically active cells, dead cells, and cells nuclei, respectively (as also described in [22]). Tumor spheroids area and live/dead cells were measured by ImageJ software.

### 2.8 Intracranial Xenografting of Fluorescent GSC#1

In vivo experiments were conducted in accordance with the institutional directives and were approved by the Italian Ministry of Health (Pr. No. 879/2021‐PR). 2 × 10^5^ GFP‐expressing‐GSC#1, resuspended in 5 μL of serum‐free DMEM, were xenografted intracranially in SCID mice (male; 4–6 week old; Charles River, Italy). For GFP lentivirus‐based transduction of GSC#1, please refer to [23], and for brain grafting refer to [24]. Briefly, GSC#1 were stably labeled by lentiviral transduction of GFP. HEK293T cells were used as packaging cell line. Cells were cotransfected by Lipofectamine reagent (Life Technologies, Waltham, MA, USA) with the lentiviral construct pCLLsin.PPT.hPGK.GFP.pre [25] together with the packaging plasmids pMDL, pRSV, and VSVG. Viral supernatant was used to perform three successive rounds of infection on GSC#1 cells. Sixteen weeks after grafting, a time when the tumor xenograft invaded the injected striatum and began to cross the corpus callosum [26], the mice were randomly divided into three groups and treated for additional 2 weeks according to the protocol 5 days on, 2 days off, as follows: axitinib was intraperitoneally injected (12.5 mg/kg of body weight); NAC was added to drinking water (1 g/kg of body weight). For axitinib and NAC concentrations, we referred to [27, 28], respectively.

### 2.9 Immunofluorescence

Tissue sections (thickness: 40 μm) were obtained from tissues embedded in OCT (Sakura Finetek, Torrance, CA, United States) using a cryostat at −20°C. Slices were treated as in [29]. Briefly, slices were blocked in PBS with 10% bovine serum albumin (BSA) and 0.3% Triton X‐100 for 45 min, before staining over night at +4°C with primary antibody in PBS with 0.3% Triton X‐100 and 0.1% normal donkey serum (NDS). The following primary antibodies were used: rat antimouse CD31 (1:100; cat no. BD 550274, clone no. MEC13.3, BD Bioscience, Franklin Lakes, NJ), rabbit antimouse Ki67 (1:100; cat no. RM9106, clone no. SP6, Thermo Scientific), mouse antihuman LMNB1 (1:100; cat no. 91251, clone no. CL3929, Atlas antibodies, Bromma, Sweden). Slices were incubated in PBS containing 0.1% NDS with the following secondary antibodies: Alexa Fluor 647 donkey antimouse, Alexa Fluor 488 or 555 donkey antirat (1:500; Thermo Fisher Scientific, Waltham, MA) for 1 h at room temperature. Lectin from Lycopersicon esculentum (tomato) biotin conjugate (1:400; cat no. L0651, Sigma‐Aldrich) was used to stain microvessels. For lectin immunostaining, sections were incubated for 1 h at room temperature in PBS containing 0.1% NDS with streptavidin Alexa Fluor 555 or Alexa Fluor 647 conjugate (1:500; Thermo Fisher Scientific). Following DAPI incubation (10 min; 1:1000; Sigma‐Aldrich), slices were mounted on glass coverslips and images were collected with TCS SP5 confocal laser scanning microscope (Leica Microsystems). Ki67‐positive cells, CD31, and Lectin staining were quantified by using QuPath software (v. 0.4.3) [30].

### 2.10 Statistical Analysis

All data presented are expressed as mean ± SD, as reported in figure legends. Significance was calculated using a two‐tailed *t‐*test. *p* < 0.05 were considered as significant in all tests.

## 3 Results

### 3.1 Axitinib Induces Senescence in GSCs and NAC Cotreatment Does Not Prevent This Induction

We preliminary addressed the ability of axitinib to limit cell growth of GSCs. Our previous studies on axitinib were developed in bulk GBM cell lines (commercially available) [16]. Here, we used two GSC lines, namely GSC#1 and GSC#61, to confirm our observations. We chose to perform the study on GSC#1 and GSC#61 since they are representative of two different GBM subtypes, proneural and mesenchymal, respectively. GSCs are considered as the most drug‐resistant cell subpopulation in GBM [31, 32] and, as expected, they showed an intrinsic higher resistance to axitinib than stable GBM cell lines, as addressed by MTS assay (Figure S1A). Axitinib failed to massively kill GSCs, and it was not possible to calculate the IC50 concentration for these cells. We performed all the in vitro experiments described onwards with the IC30 concentration, which was calculated as the concentration capable of killing the 30% of the cells compared with the control group. IC30 concentration suffices to affect phosphorylation of ERK1/2, an axitinib‐targeted downstream pathway, in both GSC#1 and GSC#61, as addressed by western blot (Figure S1B). As expected, NAC cotreatment does not affect axitinib‐dependent reduction in cell viability (Figure S1C).

**FIGURE 1:**
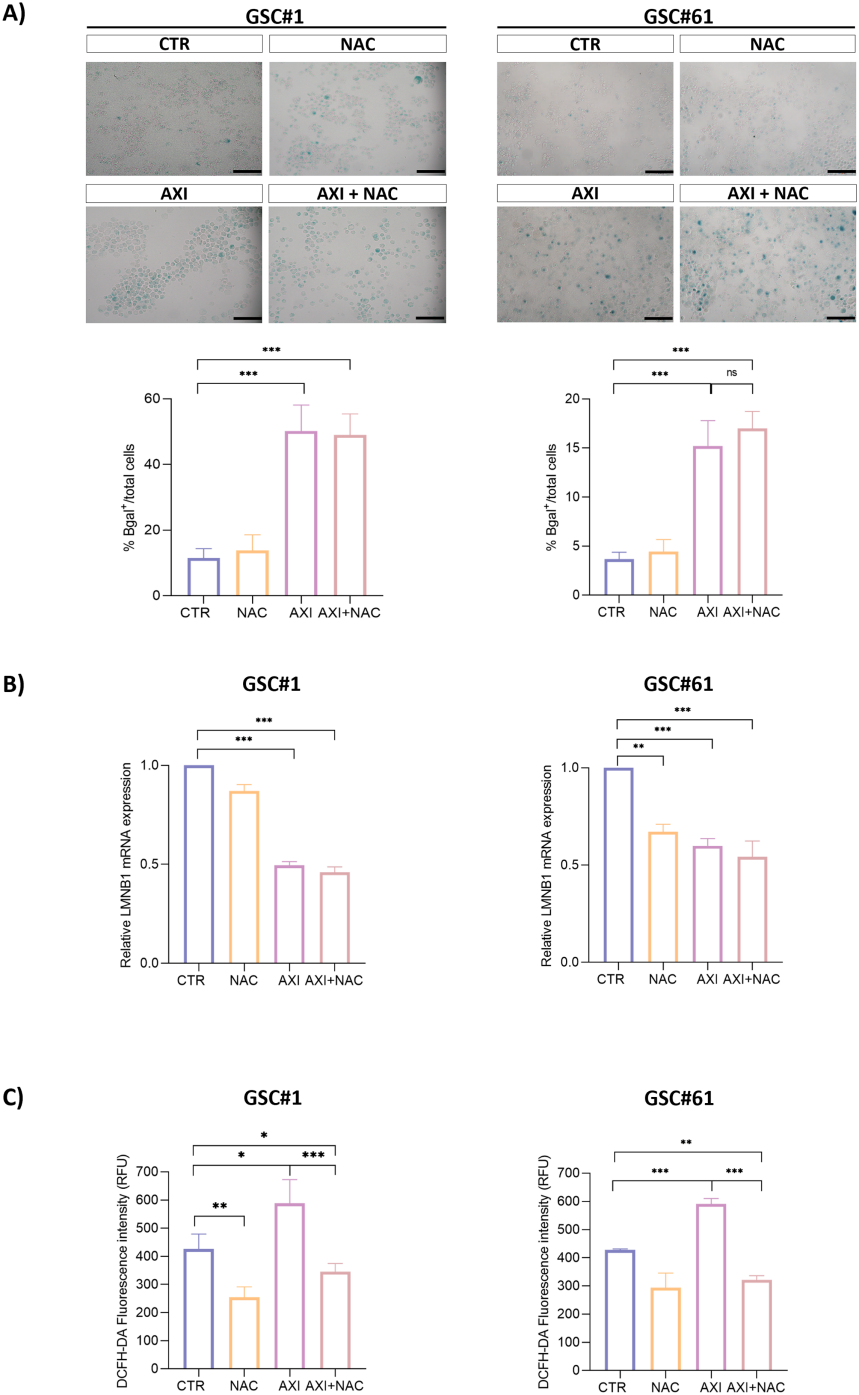
Axitinib triggers cell senescence and ROS accumulation in GSC. (A) Axitinib triggers senescence in GSC#1 and GSC#61, as addressed by SA‐β‐gal staining 4 and 7 days posttreatment, respectively. The percentage of SA‐β‐gal‐positive cells increases with axitinib and axitinib + NAC cotreatment. Magnification 20× , scale bar 100 μm. (B) Real‐time PCR experiments on GSC#1 and GSC#61 cotreated with axitinib and NAC show that ROS buffering by NAC do not prevent axitinib‐dependent gene expression change in *LMNB1*, a well‐recognized marker of cellular senescence. Relative quantities were calculated normalizing for *TBP* and are given relative to control (untreated). *n* = 3 biological replicates, *p < 0.05; **p < 0.01; ***p < 0.001. (C) GSCs were treated with axitinib or axitinib + NAC, stained with the redox‐sensitive fluorescent dye DCFHDA and analyzed by plate reader 3 days postdrug treatment. A significant increase in ROS‐associated fluorescence was observed in the presence of axitinib. NAC cotreatment protects cells from axitinib‐induced oxidative stress.

Our previous observations revealed that NAC cotreatment selectively impairs axitinib‐induced senescence in normal cells, without altering the antitumor activity on tumor cells [16]. To confirm these observations on GSCs, we characterized cellular senescence by SA‐β‐galactosidase staining and *lamin B1* expression. In agreement with previous observations on stable GBM cell lines, axitinib‐induced senescence in GSC#1 and GSC#61 (Figure 1A), and lowered the expression of *lamin B1*, whose downregulation is peculiar of senescent cells (Figure 1 B). The mechanism of senescence induction by axitinib implies ROS increase, as addressed by 2′,7′‐dichlorofluorescein diacetate labelling (Figure 1C). Strikingly, NAC cotreatment does not prevent axitinib‐induced senescence, although buffering ROS increase in GSCs (Figure 1A–C). These experiments demonstrate that, as already observed in stable GBM cell line, axitinib has a prosenescence effect on GSCs, which is paralleled by ROS increase. NAC cotreatment, although blunting ROS increase, does not prevent axitinib‐induced senescence. Overall, here we demonstrate that GSCs, as already demonstrated in bulk GBM cells, undergo senescence upon axitinib exposure. Antioxidants are not able to revert induction of cell senescence in GSCs, as opposed to what observed in endothelial cells [16].

### 3.2 Axitinib Limits the Growth of GBM Cells in 3D Tumor Spheroids in Single Treatment and in Cotreatment With NAC

We tested axitinib antitumor activity in 3D tumor spheroids established from GSC#1 and GSC#61.

**FIGURE 2:**
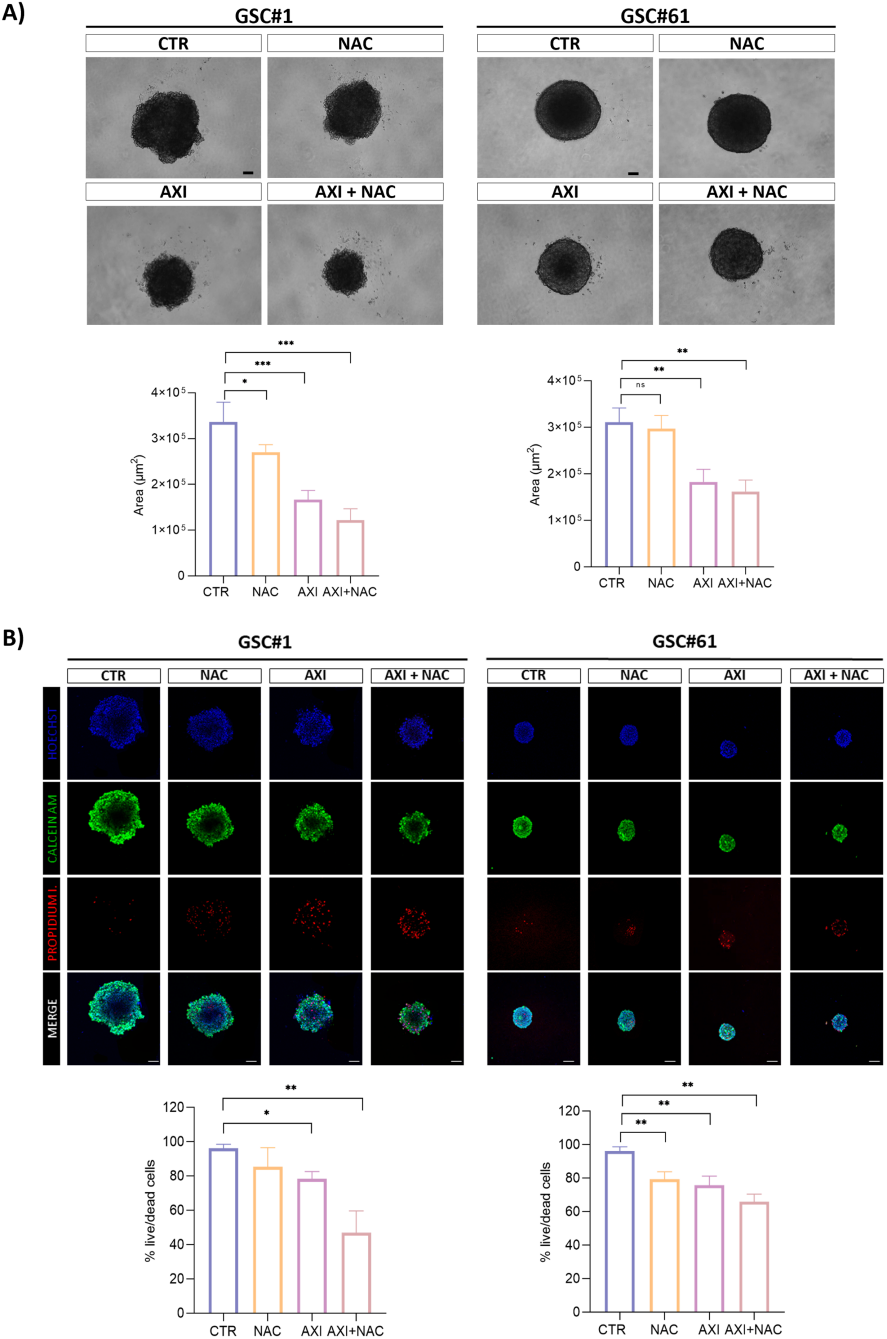
Axitinib effect on GSC‐derived 3D spheroid cultures. (A) 3D tumor spheroids were established in ultralow attachment multiwell plates (ULA) in a collagen matrix and treated for 7 days with vehicle (CTR), 5 mM NAC, 2.5 μM (GSC#1) and 10 μM (GSC#61) axitinib or with a combination of axitinib plus NAC. Axitinib significantly impairs the ability of tumor spheroids to grow. NAC cotreatment does not impair the antitumor effect of axitinib as addressed in terms of spheroid area. (B) Combined fluorescence images of GSC‐derived 3D tumor spheroids. Tumor spheroids were treated with vehicle (CTR), NAC, axitinib or axitinib‐NAC. After 7 days of treatment, tumor spheroids were stained with calcein, propidium, and Hoechst, for staining metabolically active cells, dead cells, and cell nuclei, respectively, and analyzed by confocal microscope. Overall, axitinib decreases the live/dead cells ratio. NAC cotreatment does not prevent the antitumor effect of axitinib (B). Magnification 10×, scale bar 100 μm. *n* = 10 biological replicates. *p < 0.05; **p < 0.01; ***p < 0.001.

3D cell culture models reflect some features of solid tumors, such as their architecture, secretion of soluble factors, specific gene expression panels, and drug resistance mechanisms, providing an in vitro model closer to the parental tumor than conventional flat cell cultures. In Figure 2A, the bright‐field microscopy examination of 3D GSC#1 and GSC#61 spheroids is shown. By comparing control spheroids (vehicle‐treated) with axitinib‐treated spheroids, we measured a significantly impaired tumor spheroid growth in both the cell lines tested upon axitinib exposure. Strikingly, NAC cotreatment does not affect the axitinib antitumor effect (Figure 2A). In addition, live imaging performed upon spheroid staining by calcein AM or propidium iodide, which label metabolically active and dead cells, respectively, confirmed that the axitinib antitumor effect was maintained in axitinib‐NAC cotreated tumors (Figure 2B).

### 3.3 Effects of Axitinib and Axitinib Plus NAC Treatments on Orthotopically Engrafted Mice

**FIGURE 3:**
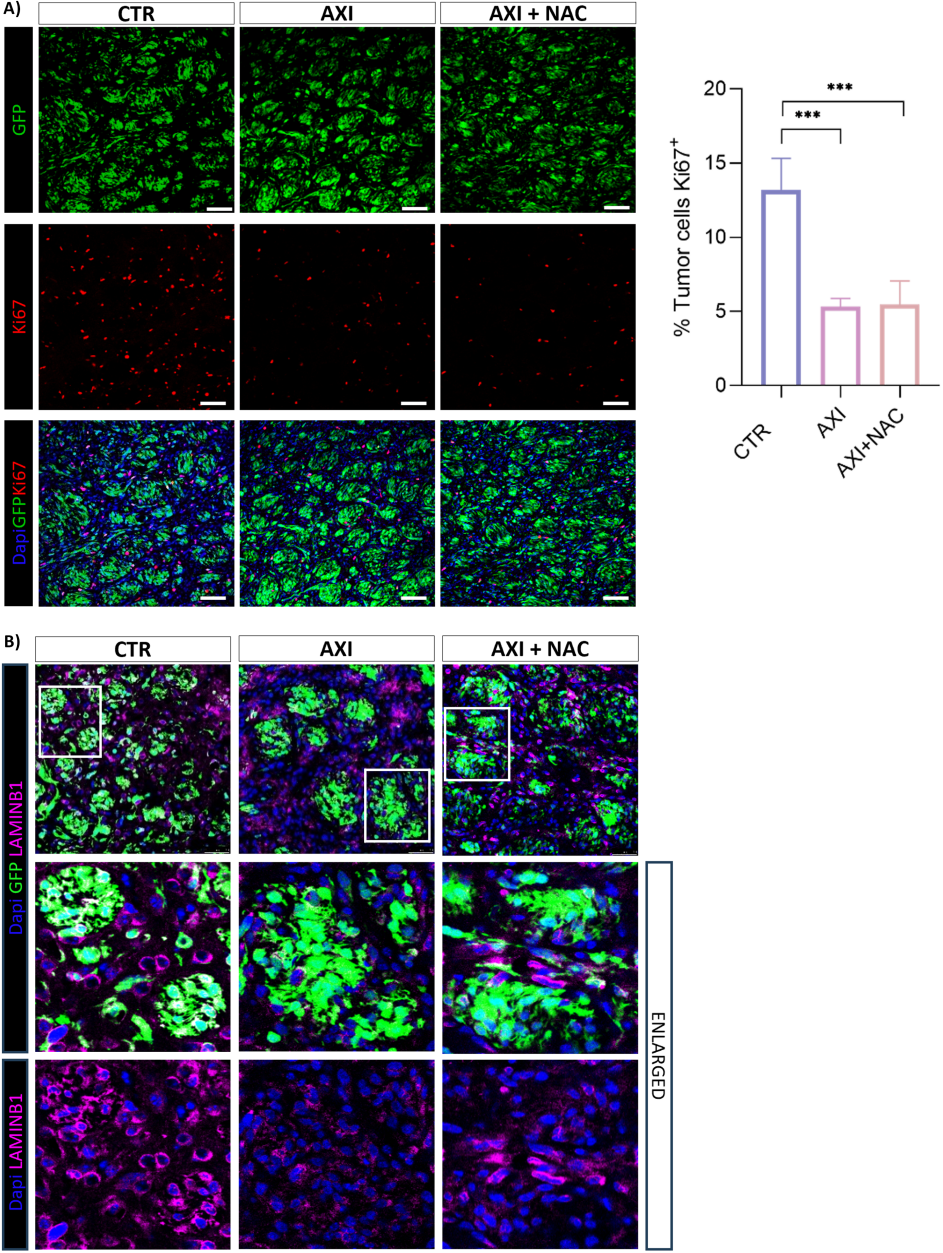
Axitinib and axitinib‐NAC treatments reduce expression of Ki67 proliferative marker and of senescence‐associated marker Lamin B1 in GSC#1 brain orthotopic xenografts. (A) Both axitinib and axitinib‐NAC treatments significantly impair the expression of the proliferative marker Ki67 and (B) of the senescence‐associated marker Lamin B1 in GFP‐expressing GSC#1 brain tumor xenografts, as addressed by immunostaining with anti Ki67 and anti‐Lamin B1 antibodies, respectively. Magnification 20×, scale bar 100 μm (A); Magnification 40×, scale bar 100 μm (B). ***p < 0.001.

**FIGURE 4:**
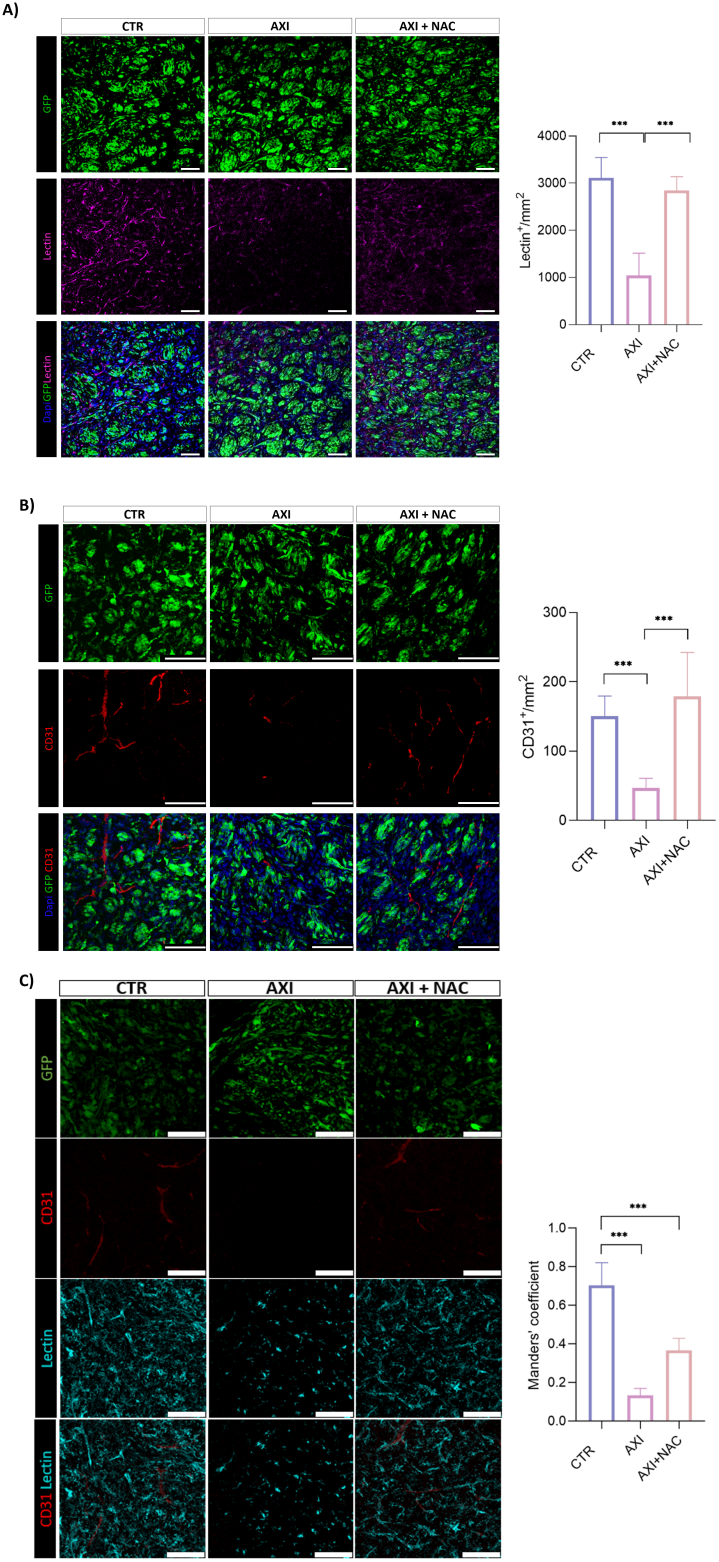
NAC rescues the axitinib‐dependent reduction in the endothelial cell markers CD31 and lectin in GSC#1 brain orthotopic xenografts. Axitinib significantly reduces the density of endothelial cells in the brain tumor tissue as addressed by lectin (A) and CD31 (B) immunostaining, respectively. Axitinib‐NAC cotreatment protects endothelial cells from axitinib‐dependent depletion, as addressed by lectin (A) and CD31 (B) immunostaining and also confirmed by CD31‐lectin costaining (C). Magnification 20×, scale bar 100 μm (A); magnification 40×, scale bar 100 μm (B) and (C). ***p < 0.001.

As previously reported, our in vivo model is based on the intracerebral injection of GSCs in immunodeficient mice eventually resulting in the development of highly infiltrative tumors mimicking the histopathological features of human malignant gliomas [20, 33]. Stably GFP‐expressing GSC#1 were grafted onto the striatum of SCID mice. Sixteen weeks after grafting, a time necessary for engrafted GSCs to develop the brain tumor, mice were divided into three experimental groups which were treated for additional 2 weeks with DMSO, as vehicle, with axitinib or with combined axitinib plus NAC, as detailed in the methods section. Immunofluorescence performed on xenografted brains revealed that in both axitinib‐ and axitinib plus NAC‐treated mice the expression of the proliferative marker Ki67 resulted significantly reduced when compared to vehicle‐treated mice (Figure 3). No significant differences in Ki67 expression between axitinib‐ and axitinib plus NAC‐treated mice were observed, coherently with our previous data [16], demonstrating that axitinib antitumor activity is not impaired in vivo as well. In addition, and again in accordance with previous in vitro observations [16], the expression of the senescence‐associated marker lamin B1 by tumor cells was reduced in axitinib‐ and in axitinib plus NAC‐treated mice. The study of brain tumor endothelium, performed by immunostaining with lectin and with anti CD31 antibody, or by coimmunostaining with lectin and CD31 again confirmed our previous in vitro observations [16]. (Figure 4A,B) shows that axitinib significantly reduces the density (cell number per mm^2^) of endothelial cells in the brain tumor tissue. Strikingly, in axitinib‐NAC experimental group, endothelial cells density is comparable to the one of the vehicle‐treated experimental group, supporting the hypothesis that NAC cotreatment exerts a protection effect on endothelial cells allowing vessel normalization. Colocalization of lectin and CD31 staining was characterized by the Manders Overlap Coefficient that allows the precise identification of the colocalization of two or more markers (Figure 4C). Again, this analysis supports the data that axitinib treatment impairs CD31/lectin costaining in blood vessels, while NAC cotreatment blunts this effect.

**FIGURE 5:**
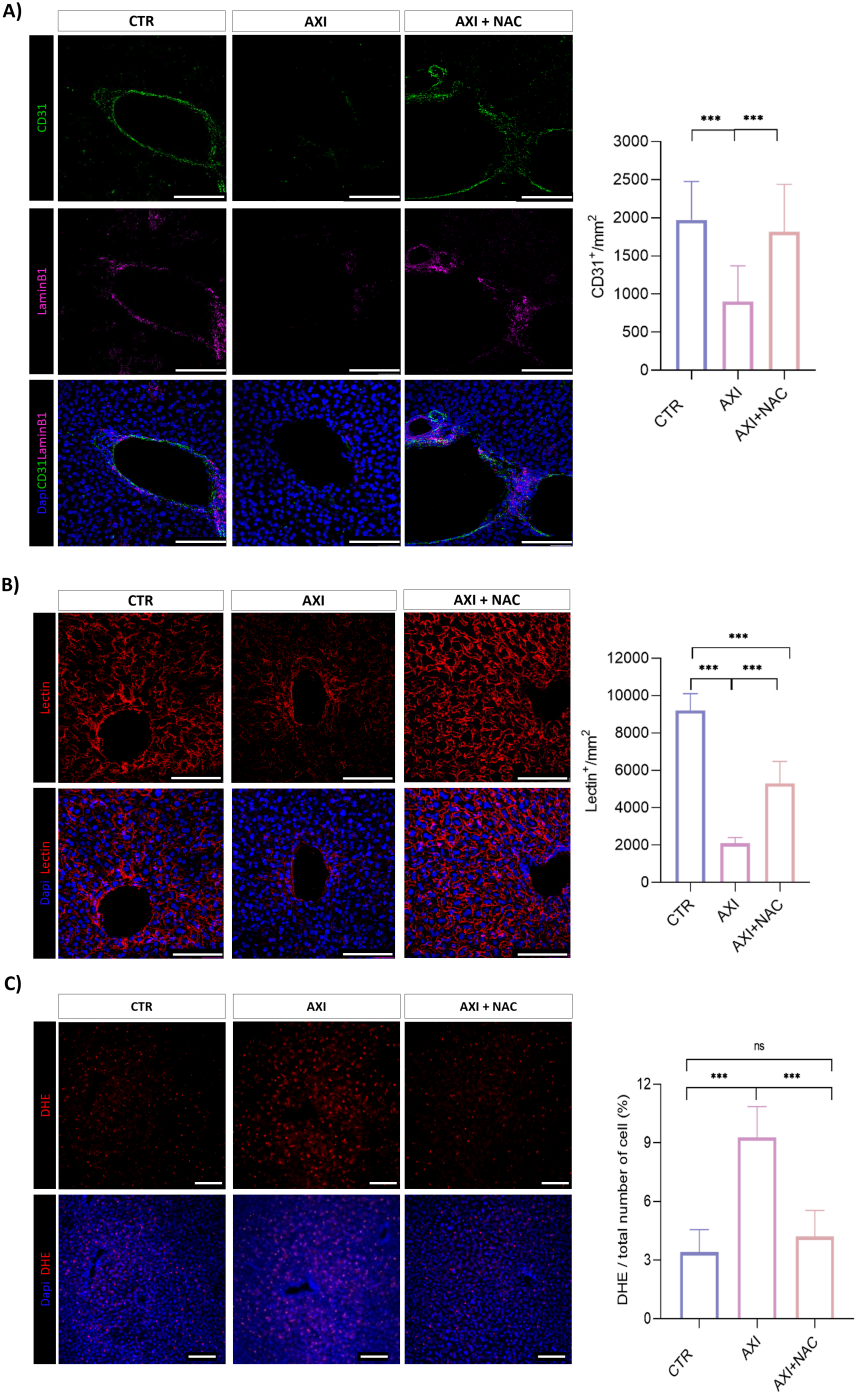
NAC cotreatment protects liver endothelium from axitinib toxicity and oxidative stress. Axitinib significantly reduces the density of endothelial cells in the liver of mice bearing brain tumor xenografts, as addressed by CD31 (A) and lectin (B) immunostaining, respectively. Magnification 40×, scale bar 100 μm. (C) NAC effectively counteracts axitinib‐induced ROS accumulation in liver tissue as addressed by DHE staining. Magnification 10×, scale bar 100 μm. ***p < 0.001.

To address if NAC exerts its protective effect from axitinib‐dependent toxicity in filter organs, we examined the liver endothelium of xenografted mice. Hepatic toxicity is indeed a quite common event in patients treated with tyrosine kinase inhibitors, including axitinib, occurring in 23%–40% of cases [34, 35]. We again focused on endothelial cells density. Liver tissue sections were immunostained with anti CD31 antibody (Figure 5A) or with lectin (Figure 5B). The density of CD31‐positive cells hugely decreases in the liver of axitinib‐treated mice. Conversely, and in accordance with data obtained in the brain, in livers from axitinib‐NAC cotreated animals the density of CD31‐positive cells is comparable to the one of vehicle‐treated animals (Figure 5A). When we stained liver vessels by lectin, again we observed a significant decrease in the number of vessels upon axitinib administration, which was, although not completely, recovered in axitinib‐NAC cotreated mice (Figure 5B). As opposed to CD31, lectin stains also liver sinusoidal endothelial cells (LSEC) [36], specialized endothelial cells representing the interface between blood cells and hepatocytes, eventually resulting in a higher percentage of stained cells. Notably, NAC confirms its protective effect from axitinib toxicity on LSEC too.

Of note, and coherently with previous in vitro observations on endothelial cells [16], axitinib promotes ROS increase which is effectively prevented in NAC cotreated mice (Figure 5C).

## 4 Discussion

In the present work we used preclinical models established from patient‐derived GSCs to confirm the previous observation that the antioxidant molecule NAC counteracts axitinib‐dependent toxicity. Overall our data (1) confirm in GSCs grown in 2D cultures and in 3D tumor spheroids the previous observation obtained in stable GBM cell lines grown in 2D [16]: axitinib limits GBM tumor cells growth. Cotreatment with NAC does not impair axitinib antitumor effectiveness; (2) in vivo, in orthotopic brain tumor xenografts obtained from GSCs engraftment, the data is confirmed as well: axitinib lowers the tumor cells proliferative index, and NAC does not affect axitinib antitumor effectiveness; (3) NAC cotreatment rescues the axitinib toxicity on tumor vasculature, promoting vessel normalization; (4) NAC cotreatment rescues axitinib‐mediated toxicity in liver, by exerting a protective action on endothelial vessels.

The data presented here confirm that normal (endothelial) and cancer (tumor stem) cells respond differently to axitinib treatment, both in vitro and in mouse models of orthotopic GBM. Axitinib, a multikinase inhibitor characterized by a pronounced specificity for VEGFR‐1, ‐2 and ‐3, has been introduced as therapeutic option for advanced renal cell carcinoma [37]. Concerning GBM, although its peculiar neo‐angiogenesis which is considered an elective therapeutic target, axitinib substantially disappointed the expectations, demonstrating a limited clinical effectiveness as a monotherapy [10, 11]. The molecular mechanism at the basis of axitinib response in GBM has not been clarified yet. We observed low levels of VEGFRs in our cells suggesting the involvement of other pathways that may act alongside the VEGF cascade andlead to ERK1/2 pathway inhibition. GBM is poorly responsive to therapy and invariably lethal. It is therefore reasonable to try to by‐pass its intractability cotargeting different pathways at the same time, with the goal to concomitantly interfere with different functions necessary for tumor development and for therapeutic response. Data have been published demonstrating that axitinib might be used in combination therapies with improved results [27]. In addition, it was reported that axitinib treatment in recurrent GBM patients is associated with increased regulatory T cell numbers and T cell exhaustion, suggesting that axitinib treatment in patients with recurrent GBM has a favorable impact on immune function [12]. The relevance of considering the possibility of a combination therapy for axitinib and the role of axitinib as an enhancer of immune response were both confirmed by Chryplewicz and colleagues in a very interesting recent work [13]. Authors describe the striking anticancer effects of using the tricyclic antidepressant imipramine together with axitinib in preclinical models of GBM. This synergistic effect is mainly due to autophagy increase in cancer cells together with a reprogram of tumor‐associated macrophage phenotype, and a modified vessel network.

Finally, a key implication of our data is related to vessel normalization promoted by NAC in the tumor vasculature of xenografted mice. As recently discussed in [38], hypoxia negatively affects cancer prognosis lowering the activation and infiltration of cells of the immune system in the tumor microenvironment. Vessel normalization of the aberrant GBM vasculature, as obtained by NAC, besides allowing a more efficient drug delivery to the tumor mass, might eventually result in hypoxia reduction and in the promotion of immune cells activation.

In the key of a combination therapy, although the safety of NAC cotreatment in humans needs to be investigated, axitinib can now be seen in a new perspective and our data contribute to empower the possibility of axitinib use for GBM patients' therapy.

## Author Contributions

**Alessia Formato:** data curation (equal), formal analysis (equal), investigation (equal), visualization (equal). **Maria Salbini:** data curation (equal), formal analysis (equal), investigation (equal), visualization (equal). **Elisa Orecchini:** data curation (supporting), investigation (supporting). **Manuela Pellegrini:** data curation (equal), funding acquisition (equal). **Mariachiara Buccarelli:** investigation (supporting). **Lucia Ricci Vitiani:** data curation (supporting). **Stefano Giannetti:** investigation (supporting). **Roberto Pallini:** conceptualization (supporting), funding acquisition (lead), writing – review and editing (supporting). **Quintino Giorgio D'Alessandris:** conceptualization (supporting), investigation (supporting). **Liverana Lauretti:** investigation (supporting). **Maurizio Martini:** investigation (supporting). **Valentina De Falco:** methodology (equal), validation (equal). **Andrea Levi:** data curation (supporting), writing – review and editing (supporting). **Maria Laura Falchetti:** conceptualization (equal), funding acquisition (lead), project administration (lead), supervision (equal), visualization (equal), writing – original draft (lead). **Maria Patrizia Mongiardi:** conceptualization (equal), funding acquisition (equal), investigation (equal), project administration (supporting), supervision (lead), writing – review and editing (supporting).

## Ethics Statement

This study was approved by the Italian Ministry of Health (Prot. No. 879/2021‐PR). All patients signed an informed consent form allowing for collection of tumor samples and clinical data and for cell line generation.

## Conflicts of Interest

The authors declare no conflicts of interest.

## Supporting information


**Figure S1:** (A) Cell viability assay of axitinib and axitinib+NAC‐treated cells. GSC#1, GSC#61 and HeLa, as a non‐GBM tumor cell line, were vehicle‐treated (CTR) or treated with axitinib concentrations in the range 1–20 μM for 48 h before cell viability assay (MTS). (B) Protein expression of ERK 1/2 in Axitinib‐treated GBM cells. GSC#1, and GSC#61 were treated with axitinib (IC30) for 24, 4 and 1 h, respectively. Figure shows a representative western blot analysis of total and phosphorylated ERK 1/2, analyzed as an indirect strategy to confirm axitinib effectiveness in our cells. Vinculin was used as protein‐loading control. (C) GSC#1, GSC#61 and HeLa were vehicle‐treated (CTR) or treated with axitinib (IC30), NAC (5 mM) or axitinib+NAC for 48 h, stained with calcein AM and analyzed by flow cytometer to assess cell viability. The histogram shows the percentage of calcein AM‐positive cells normalized on the number of cells for each experimental condition. *n* = 3 biological replicates; **p < 0.01; ***p < 0.001.

## Data Availability

The data that support the findings of this study are available from the corresponding author upon reasonable request.

## References

[1] M. Gross‐Goupil , L. François , A. Quivy , and A. Ravaud , “Axitinib: A Review of Its Safety and Efficacy in the Treatment of Adults With Advanced Renal Cell Carcinoma,” Clinical Medicine Insights 7 (2013): 269–277. 24250243 10.4137/CMO.S10594 PMC3825605

[2] D. D. Hu‐Lowe , H. Y. Zou , M. L. Grazzini , et al., “Nonclinical Antiangiogenesis and Antitumor Activities of Axitinib (AG‐013736), an Oral, Potent, and Selective Inhibitor of Vascular Endothelial Growth Factor Receptor Tyrosine Kinases 1, 2, 3,” Clinical Cancer Research 14 (2008): 7272–7283. 19010843 10.1158/1078-0432.CCR-08-0652

[3] A. Bellesoeur , E. Carton , J. Alexandre , F. Goldwasser , and O. Huillard , “Axitinib in the Treatment of Renal Cell Carcinoma: Design, Development, and Place in Therapy,” Drug Design, Development and Therapy 11 (2017): 2801–2811. 29033542 10.2147/DDDT.S109640 PMC5614734

[4] J. R. Infante , T. R. Reid , A. L. Cohn , et al., “Axitinib and/or Bevacizumab With Modified FOLFOX‐6 as First‐Line Therapy for Metastatic Colorectal Cancer: A Randomized Phase 2 Study,” Cancer 119 (2013): 2555–2563. 23605883 10.1002/cncr.28112

[5] J. C. Bendell , M. Joseph , K. Barnes , et al., “A Phase‐2 Trial of Single Agent Axitinib as Maintenance Therapy Following First‐Line Treatment With Modified FOLFOX/Bevacizumab in Patients With Metastatic Colorectal Cancer,” Cancer Investigation 35 (2017): 386–392. 28426267 10.1080/07357907.2017.1310221

[6] D. N. Louis , A. Perry , P. Wesseling , et al., “The 2021 WHO Classification of Tumors of the Central Nervous System: A Summary,” Neuro‐Oncology 23 (2021): 1231–1251. 34185076 10.1093/neuonc/noab106 PMC8328013

[7] O. L. Chinot , W. Wick , W. Mason , et al., “Bevacizumab Plus Radiotherapy‐Temozolomide for Newly Diagnosed Glioblastoma,” New England Journal of Medicine 370 (2014): 709–722. 24552318 10.1056/NEJMoa1308345

[8] D. A. Reardon , S. Turner , K. B. Peters , et al., “A Review of VEGF/VEGFR‐Targeted Therapeutics for Recurrent Glioblastoma,” Journal of the National Comprehensive Cancer Network 9 (2011): 414–427. 21464146 10.6004/jnccn.2011.0038 PMC3399727

[9] P. S. Zeiner , M. Kinzig , I. Divé , et al., “Regorafenib CSF Penetration, Efficacy, and MRI Patterns in Recurrent Malignant Glioma Patients,” Journal of Clinical Medicine 8 (2019): 8. 10.3390/jcm8122031 PMC6947028 31766326

[10] J. Duerinck , S. Du Four , F. Bouttens , et al., “Randomized Phase II Trial Comparing Axitinib With the Combination of Axitinib and Lomustine in Patients With Recurrent Glioblastoma,” Journal of Neuro‐Oncology 136 (2018): 115–125. 28988341 10.1007/s11060-017-2629-z

[11] J. Duerinck , S. Du Four , F. Vandervorst , et al., “Randomized Phase II Study of Axitinib Versus Physicians Best Alternative Choice of Therapy in Patients With Recurrent Glioblastoma,” Journal of Neuro‐Oncology 128 (2016): 147–155. 26935577 10.1007/s11060-016-2092-2

[12] S. Du Four , S. K. Maenhout , D. Benteyn , et al., “Disease Progression in Recurrent Glioblastoma Patients Treated With the VEGFR Inhibitor Axitinib is Associated With Increased Regulatory T Cell Numbers and T Cell Exhaustion,” Cancer Immunology, Immunotherapy 65 (2016): 727–740. 27098427 10.1007/s00262-016-1836-3 PMC11029796

[13] A. Chryplewicz , J. Scotton , M. Tichet , et al., “Cancer Cell Autophagy, Reprogrammed Macrophages, and Remodeled Vasculature in Glioblastoma Triggers Tumor Immunity,” Cancer Cell 40 (2022): 1111–1127.e1119. 36113478 10.1016/j.ccell.2022.08.014 PMC9580613

[14] M. B. Morelli , C. Amantini , M. Santoni , et al., “Axitinib Induces DNA Damage Response Leading to Senescence, Mitotic Catastrophe, and Increased NK Cell Recognition in Human Renal Carcinoma Cells,” Oncotarget 6 (2015): 36245–36259. 26474283 10.18632/oncotarget.5768 PMC4742174

[15] M. B. Morelli , C. Amantini , M. Nabissi , et al., “Axitinib Induces Senescence‐Associated Cell Death and Necrosis in Glioma Cell Lines: The Proteasome Inhibitor, Bortezomib, Potentiates Axitinib‐Induced Cytotoxicity in a p21(Waf/Cip1) Dependent Manner,” Oncotarget 8 (2017): 3380–3395. 27926485 10.18632/oncotarget.13769 PMC5356889

[16] M. P. Mongiardi , G. Radice , M. Piras , et al., “Axitinib Exposure Triggers Endothelial Cells Senescence Through ROS Accumulation and ATM Activation,” Oncogene 38 (2019): 5413–5424. 30967634 10.1038/s41388-019-0798-2

[17] G. Marziali , M. Signore , M. Buccarelli , et al., “Metabolic/Proteomic Signature Defines Two Glioblastoma Subtypes With Different Clinical Outcome,” Scientific Reports 6 (2016): 21557. 26857460 10.1038/srep21557 PMC4746700

[18] R. Pallini , L. Ricci‐Vitiani , G. L. Banna , et al., “Cancer Stem Cell Analysis and Clinical Outcome in Patients With Glioblastoma Multiforme,” Clinical Cancer Research 14 (2008): 8205–8212. 19088037 10.1158/1078-0432.CCR-08-0644

[19] R. Galli , E. Binda , U. Orfanelli , et al., “Isolation and Characterization of Tumorigenic, Stem‐Like Neural Precursors From Human Glioblastoma,” Cancer Research 64 (2004): 7011–7021. 15466194 10.1158/0008-5472.CAN-04-1364

[20] A. Eramo , L. Ricci‐Vitiani , A. Zeuner , et al., “Chemotherapy Resistance of Glioblastoma Stem Cells,” Cell Death and Differentiation 13 (2006): 1238–1241. 16456578 10.1038/sj.cdd.4401872

[21] R. Lucà , M. R. Assenza , F. Maiullari , et al., “Inhibition of the mTOR Pathway and Reprogramming of Protein Synthesis by MDM4 Reduce Ovarian Cancer Metastatic Properties,” Cell Death & Disease 12 (2021): 558. 34052831 10.1038/s41419-021-03828-z PMC8164635

[22] M. P. Mongiardi , M. Buccarelli , A. Formato , et al., “Characterization of Glioblastoma Cells Response to Regorafenib,” Cancers (Basel) 24 (2022): 14. 10.3390/cancers14246193 PMC9777191 36551679

[23] S. Pacioni , Q. G. D'Alessandris , S. Giannetti , et al., “Mesenchymal Stromal Cells Loaded With Paclitaxel Induce Cytotoxic Damage in Glioblastoma Brain Xenografts,” Stem Cell Research & Therapy 6 (2015): 194. 26445228 10.1186/s13287-015-0185-z PMC4594910

[24] M. L. Falchetti , Q. G. D'Alessandris , S. Pacioni , et al., “Glioblastoma Endothelium Drives Bevacizumab‐Induced Infiltrative Growth via Modulation of PLXDC1,” International Journal of Cancer 144 (2019): 1331–1344. 30414187 10.1002/ijc.31983 PMC6590500

[25] T. Dull , R. Zufferey , M. Kelly , et al., “A Third‐Generation Lentivirus Vector With a Conditional Packaging System,” Journal of Virology 72 (1998): 8463–8471. 9765382 10.1128/jvi.72.11.8463-8471.1998 PMC110254

[26] T. L. Haas , M. R. Sciuto , L. Brunetto , et al., “Integrin α7 is a Functional Marker and Potential Therapeutic Target in Glioblastoma,” Cell Stem Cell 21 (2017): 35–50.e39. 28602620 10.1016/j.stem.2017.04.009

[27] D. Saha , H. Wakimoto , C. W. Peters , S. J. Antoszczyk , S. D. Rabkin , and R. L. Martuza , “Combinatorial Effects of VEGFR Kinase Inhibitor Axitinib and Oncolytic Virotherapy in Mouse and Human Glioblastoma Stem‐Like Cell Models,” Clinical Cancer Research 24 (2018): 3409–3422. 29599413 10.1158/1078-0432.CCR-17-1717 PMC6050085

[28] A. Berry , V. Bellisario , P. Panetta , et al., “Administration of the Antioxidant N‐Acetyl‐Cysteine in Pregnant Mice Has Long‐Term Positive Effects on Metabolic and Behavioral Endpoints of Male and Female Offspring Prenatally Exposed to a High‐Fat Diet,” Frontiers in Behavioral Neuroscience 12 (2018): 48. 29599711 10.3389/fnbeh.2018.00048 PMC5862866

[29] S. Pacioni , Q. G. D'Alessandris , S. Giannetti , et al., “Human Mesenchymal Stromal Cells Inhibit Tumor Growth in Orthotopic Glioblastoma Xenografts,” Stem Cell Research & Therapy 8 (2017): 53. 28279193 10.1186/s13287-017-0516-3 PMC5345323

[30] P. Bankhead , M. B. Loughrey , J. A. Fernández , et al., “QuPath: Open Source Software for Digital Pathology Image Analysis,” Scientific Reports 7 (2017): 16878. 29203879 10.1038/s41598-017-17204-5 PMC5715110

[31] J. Zhao , “Cancer Stem Cells and Chemoresistance: The Smartest Survives the Raid,” Pharmacology & Therapeutics 160 (2016): 145–158. 26899500 10.1016/j.pharmthera.2016.02.008 PMC4808328

[32] A. L. V. Alves , I. N. F. Gomes , A. C. Carloni , et al., “Role of Glioblastoma Stem Cells in Cancer Therapeutic Resistance: A Perspective on Antineoplastic Agents From Natural Sources and Chemical Derivatives,” Stem Cell Research & Therapy 12 (2021): 206. 33762015 10.1186/s13287-021-02231-x PMC7992331

[33] Q. G. D'Alessandris , M. Biffoni , M. Martini , et al., “The Clinical Value of Patient‐Derived Glioblastoma Tumorspheres in Predicting Treatment Response,” Neuro‐Oncology 19 (2017): 1097–1108. 28204560 10.1093/neuonc/now304 PMC5737323

[34] R. Iacovelli , A. Palazzo , G. Procopio , et al., “Incidence and Relative Risk of Hepatic Toxicity in Patients Treated With Anti‐Angiogenic Tyrosine Kinase Inhibitors for Malignancy,” British Journal of Clinical Pharmacology 77 (2014): 929–938. 23981115 10.1111/bcp.12231 PMC4093918

[35] B. I. Rini , M. B. Atkins , E. R. Plimack , et al., “Characterization and Management of Treatment‐Emergent Hepatic Toxicity in Patients With Advanced Renal Cell Carcinoma Receiving First‐Line Pembrolizumab Plus Axitinib. Results From the KEYNOTE‐426 Trial,” European Urology Oncology 5 (2022): 225–234. 34244116 10.1016/j.euo.2021.05.007

[36] L. D. DeLeve and A. C. Maretti‐Mira , “Liver Sinusoidal Endothelial Cell: An Update,” Seminars in Liver Disease 37 (2017): 377–387. 29272898 10.1055/s-0037-1617455 PMC6005648

[37] T. Tyler , “Axitinib: Newly Approved for Renal Cell Carcinoma,” Journal of the Advanced Practitioner in Oncology 3 (2012): 333–335. 25031963 10.6004/jadpro.2012.3.5.7 PMC4093354

[38] P. Fan , N. Zhang , E. Candi , et al., “Alleviating Hypoxia to Improve Cancer Immunotherapy,” Oncogene 42 (2023): 3591–3604. 37884747 10.1038/s41388-023-02869-2

