# Supplementary figures and images for: N‐Acetyl‐L‐Cysteine (NAC) Blunts Axitinib‐Related Adverse Effects in Preclinical Models of Glioblastoma
Source: Cancer Med. 2024 Oct 8;13(19):e70279. doi: 10.1002/cam4.70279 (PMC11460215; doi:10.1002/cam4.70279)

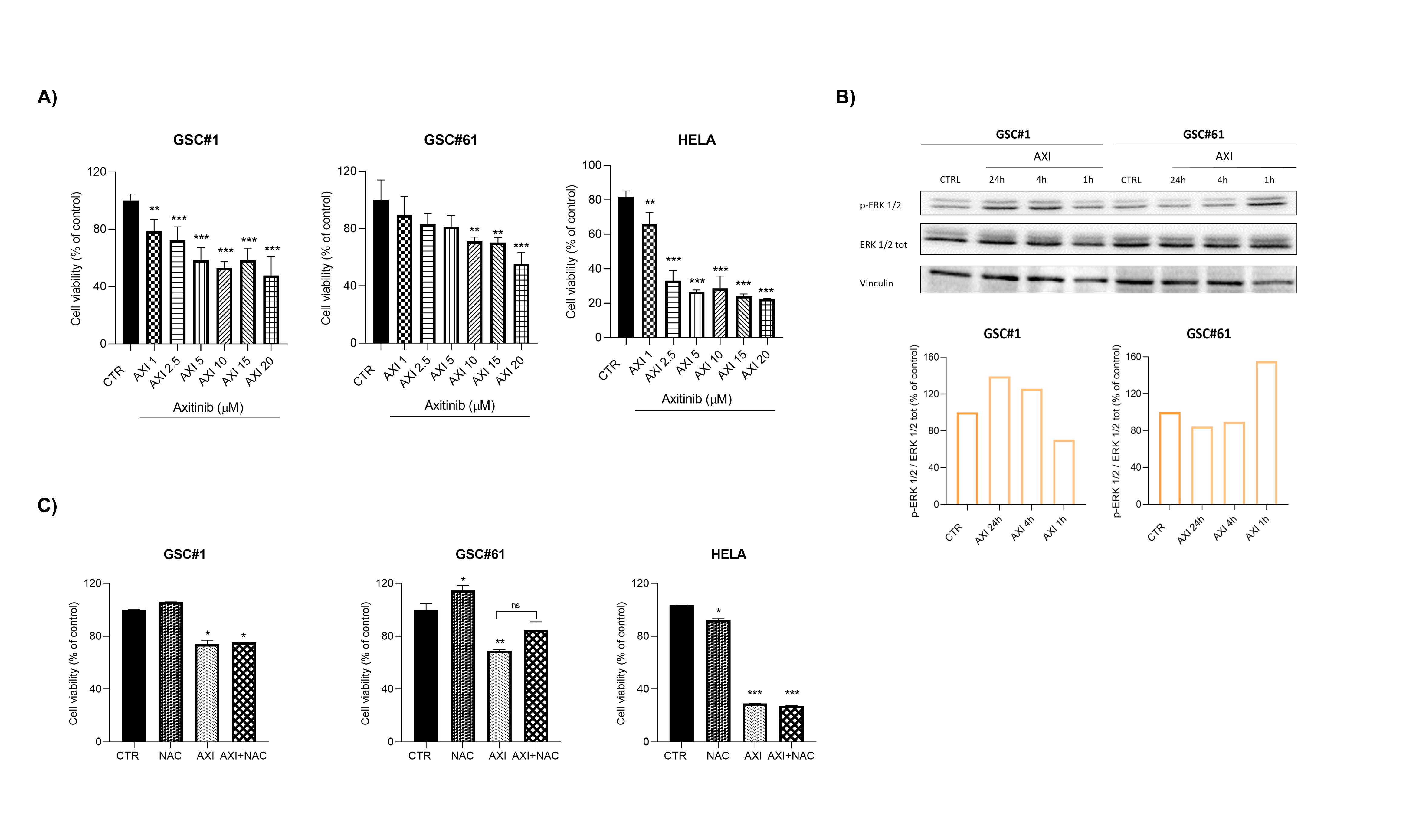

Supplement: Supplementary file 1 — Figure S1: (A) Cell viability assay of axitinib and axitinib+NAC‐treated cells. GSC#1, GSC#61 and HeLa, as a non‐GBM tumor cell line, were vehicle‐treated (CTR) or treated with axitinib concentrations in the range 1–20 μM for 48 h before cell viability assay (MTS). (B) Protein expression of ERK 1/2 in Axitinib‐treated GBM cells. GSC#1, and GSC#61 were treated with axitinib (IC30) for 24, 4 and 1 h, respectively. Figure shows a representative western blot analysis of total and phosphorylated ERK 1/2, analyzed as an indirect strategy to confirm axitinib effectiveness in our cells. Vinculin was used as protein‐loading control. (C) GSC#1, GSC#61 and HeLa were vehicle‐treated (CTR) or treated with axitinib (IC30), NAC (5 mM) or axitinib+NAC for 48 h, stained with calcein AM and analyzed by flow cytometer to assess cell viability. The histogram shows the percentage of calcein AM‐positive cells normalized on the number of cells for each experimental condition. n = 3 biological replicates; **p < 0.01; ***p < 0.001. [file CAM4-13-e70279-s001.tif]
